# Identifying Hypoxia Characteristics to Stratify Prognosis and Assess the Tumor Immune Microenvironment in Renal Cell Carcinoma

**DOI:** 10.3389/fgene.2021.606816

**Published:** 2021-06-14

**Authors:** Zhenan Zhang, Qinhan Li, Feng Wang, Binglei Ma, Yisen Meng, Qian Zhang

**Affiliations:** ^1^Department of Urology, Peking University First Hospital, Beijing, China; ^2^National Research Center for Genitourinary Oncology, Institute of Urology, Peking University, Beijing, China; ^3^Department of Urology, People's Hospital of Tibet Autonomous Region, Lhasa, China

**Keywords:** renal cell carcinoma, immune response, tumor microenvironment, hypoxia, risk model

## Abstract

**Background:** Renal cell carcinoma (RCC) is a common malignant tumor worldwide, and immune checkpoint inhibitors are a new therapeutic option for metastatic RCC. Infiltrating immune cells in the tumor microenvironment (TME) play a critical part in RCC biology, which is important for tumor therapy and prediction. Hypoxia is a common condition that occurs in the TME and may lead to RCC immunosuppression and immune escape. This study was conducted to analyze the extent of the hypoxia immune microenvironment in the TME of RCC and develop a hypoxia-related risk model for predicting the prognosis of patients with RCC.

**Methods:** The gene expression profiles of 526 patients with RCC were downloaded from The Cancer Genome Atlas database. Combined with the hallmark-hypoxia gene dataset downloaded from Gene Set Enrichment Analysis, prognosis-related hypoxia genes were selected by survival analysis. A protein–protein interaction network and functional enrichment analysis were performed. A hypoxia-related risk model predicting the prognosis of patients with RCC was established using the least absolute shrinkage and selection operator. Data of 91 cases downloaded from the International Cancer Genome Consortium (ICGC) database were used for validation. CIBERSORT was applied to analyze the fractions of 22 immune cell types in the TME of RCC between low- and high-risk groups. The expression profiles of immunomodulators and immunosuppressive cytokines were also analyzed.

**Results:** Ninety-three genes were significantly associated with poor overall survival of patients with RCC and were mainly involved in 10 pathways. Using the established hypoxia-related risk model, the receiver operating characteristic curves showed an accuracy of 76.1% (95% CI: 0.719–0.804), and Cox proportional hazards regression analysis revealed that the model was an independent predictor of the prognosis of patients with RCC [hazard ratio (HR) = 2.884; 95% CI: 2.090–3.979] (*p* < 0.001). Using the ICGC database, we verified that the low-risk score group had a better overall survival outcome than the high-risk group. Additionally, dividing the hypoxia risk score into high-risk and low-risk groups could predict the immune microenvironment of RCC.

**Conclusions:** We demonstrated that a hypoxia-related risk model can be used to predict the outcomes of patients with RCC and reflect the immune microenvironment of RCC, which may help improve the overall clinical response to immune checkpoint inhibitors.

## Introduction

Kidney cancer is a common malignant tumor worldwide, with an estimated 403,000 new cases and 175,000 deaths in 2018 (Bray et al., 2018). Renal cell carcinoma (RCC) is the most common form of kidney cancer, and ~70% of these cases show clear-cell tumors in histological analysis (Lipworth et al., 2016). Surgical resection, including radical nephrectomy and nephron-sparing surgery, remains the most effective therapy for clinically localized RCC. Once metastasis of RCC occurs, clinical treatment is challenging and patients show a 5-year survival rate of approximately 12% (Siegel et al., 2017). Cytokines [interferon (IFN)-α, interleukin (IL)-2], targeted therapy [tyrosine kinase inhibitors, anti-vascular endothelial growth factor (VEGF) antibodies, agents targeting the mammalian target of rapamycin (mTOR)], and immune checkpoint inhibitors are used as therapies for metastatic RCC. However, it is important to elevate the overall clinical response rate of cancer immunotherapy and identify biomarkers for response prediction.

Multiple factors contribute to cancer initiation and progression. The tumor microenvironment (TME) is an important regulator of tumor progression and metastasis (McAllister and Weinberg, 2014). Infiltrating immune cells are among the major normal cells in tumor tissues and play a crucial role in tumor biology, tumor prognosis, drug resistance, and immunotherapeutic efficacy (Straussman et al., 2012; van Dijk et al., 2019; Guo et al., 2020). A better understanding of the TME, particularly infiltrating immune cells, is important for improving tumor therapy and tumor prediction.

Hypoxia is a common condition found in the TME, playing a vital role in tumor genetic instability and prognosis (LaGory and Giaccia, 2016). The hypoxia-inducible transcription factor (HIF) signaling pathway can be activated by tumor-induced hypoxia (Fallah and Rini, 2019). In clear-cell RCC (ccRCC), HIF is particularly important, with HIF-1α and HIF-2α exerting opposing effects on tumor development (Schödel et al., 2016). Small-molecule inhibitors of HIF-2 may serve as another therapeutic option for ccRCC in the future (Martínez-Sáez et al., 2017). Hypoxia can lead to tumor immunosuppression and immune escape. It has been reported that hypoxia promotes suppressive immune cells and immunosuppressive cytokines in the TME (Terry et al., 2017). Therefore, hypoxia-related genes may be useful for predicting immunotherapy outcomes.

**Figure 1:**
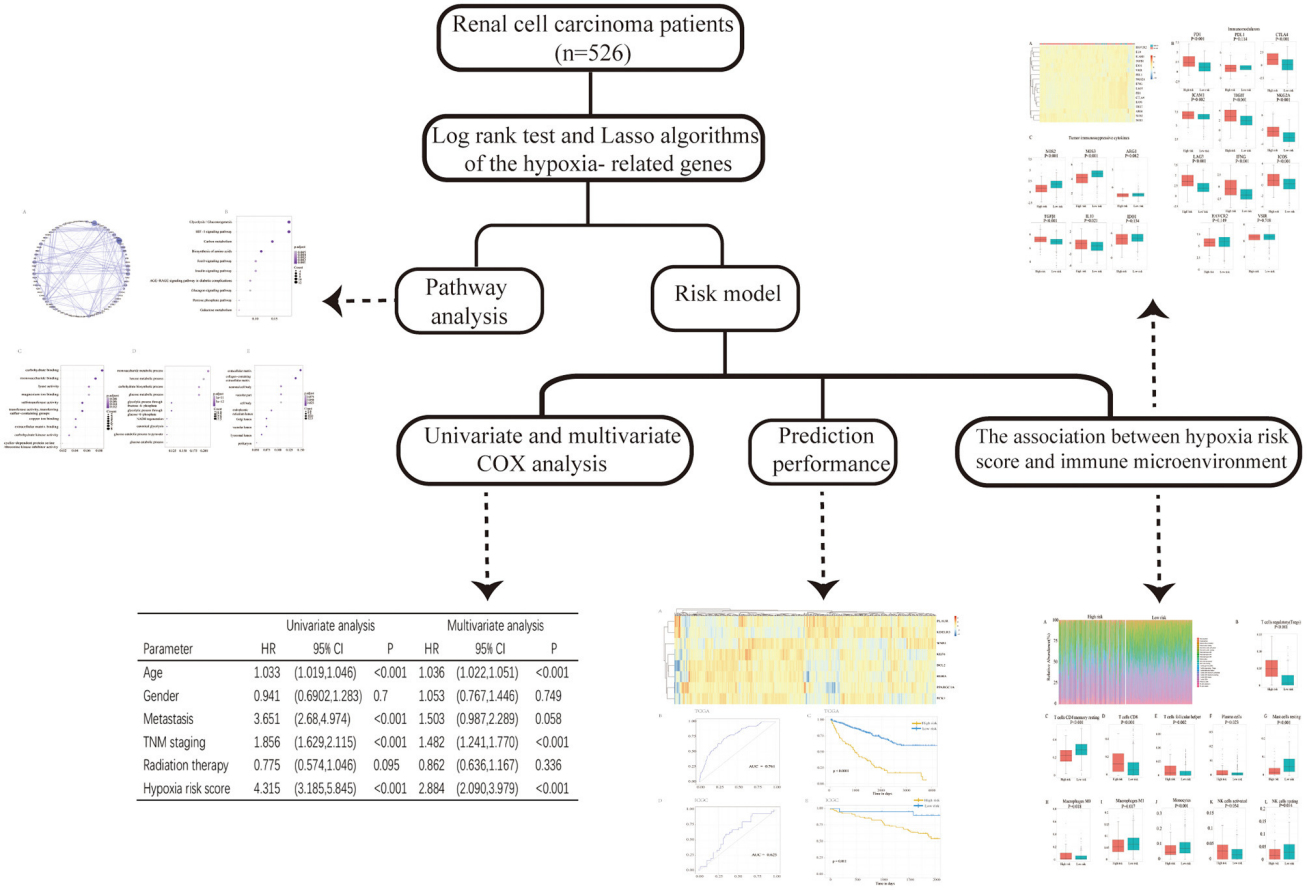
The flowchart of the study.

This study was conducted to analyze the gene expression profiles of RCC downloaded from The Cancer Genome Atlas (TCGA) database and hypoxia-related genes (hallmark-hypoxia genes) downloaded from Gene Set Enrichment Analysis (GSEA). We selected prognosis-related hypoxia genes to develop a hypoxia-related risk model for predicting the prognosis and immune microenvironment landscape of patients with RCC in high/low hypoxia risk score groups. The workflow of the study design is shown in Figure 1.

## Materials and Methods

### Database

The level 3 gene expression profiles of 526 patients with RCC were downloaded from TCGA database (https://tcga-data.nci.nih.gov/) (June 2020). The patients' clinical characteristics, including age, sex, TNM stage, and survival data, were also obtained from the database. Patients with cancer without pathologic diagnosis or a lack of clinical information were excluded.

Hypoxia-related genes (hallmark- hypoxia genes) were downloaded from GSEA (https://www.gsea-msigdb.org/gsea/index.jsp). The gene expression profiles of 91 patients with RCC determined by the CAGEKID consortium in Europe were downloaded from the International Cancer Genome Consortium (ICGC) database (https://icgc.org/icgc/cgp/65/812/817) and used as the validation cohort to verify the predictive value of the risk model.

### Construction of Protein–Protein Interaction Network and Functional Enrichment Analysis

Hypoxia genes were selected using the log-rank test to identify statistically significant prognosis-related genes. The selected hypoxia genes were used to establish a protein–protein interaction (PPI) network and for functional enrichment analysis. The Search Tool for the Retrieval of Interacting Genes (STRING) database was used to generate the PPI network (Szklarczyk et al., 2015). Thereafter, Cytoscape software (version 3.7.0) was used to reconstruct and visualize the PPI network (Shannon et al., 2003). The connectivity degree of each protein node was calculated. The R package clusterprofile was utilized to perform functional enrichment analysis (Yu et al., 2012). Based on Gene Ontology (GO) categories, the genes were identified with different GO terms based on their respective characteristics: molecular functions (MFs), biological processes (BPs), and cellular components (CCs). Additionally, Kyoto Encyclopedia of Genes and Genomes (KEGG) pathways were used for pathway enrichment analysis. The false discovery rate (FDR) was set at 0.05.

### Construction of a Risk Model

The selected prognosis-related hypoxia genes were applied in the least absolute shrinkage and selection operator (LASSO) using the R package glmnet. The hypoxia risk score formula was established based on gene expression multiplied by a linear combination of the regression coefficient, which was acquired from LASSO. The cases were divided into high- and low-risk groups based on the optimal cutoff point of the risk score with the R package survminer (version 0.4.6). R package survival and ROCR were utilized for Kaplan–Meier analysis and to generate receiver operating characteristic (ROC) curves. To draw heat maps, pheatmap (version 1.64.0) was used in R package. The predictive value of the risk model was verified using data from 91 patients with RCC downloaded from the ICGC database.

### Assessment of Immune Cell Type Fractions

Using gene expression data, the analytical method CIBERSORT (https://cibersort.stanford.edu/) can be applied to characterize the cell composition in a mixed cell population (Newman et al., 2015). The leukocyte gene signature matrix containing 547 genes, named LM22 in CIBERSORT, was applied to distinguish 22 immune cell types including CD8 T cells, naive CD4 T cells, resting memory CD4 T cells, activated memory CD4 T cells, naive B cells, memory B cells, plasma cells, follicular helper T cells, T-regulatory cells (Tregs), gamma delta T cells, resting natural killer cells, activated natural killer cells, monocytes, macrophages M0, macrophages M1, M2, resting dendritic cells, activated dendritic cells, resting mast cells, activated mast cells, eosinophils, and neutrophils. We applied CIBERSORT to assess the fractions of these cell types between the low- and high-risk groups.

### Expression Profile of Immunomodulators and Immunosuppressive Cytokines

Several key immunomodulators, including lymphocyte activation gene 3 (LAG-3), T cell immunoglobulin and mucin domain containing 3 (TIM-3), cytotoxic T lymphocyte associated protein 4 (CTLA-4), IFN-γ, ICOS inducible T cell costimulator (ICOS), intercellular adhesion molecule 1 (ICAM-1), T cell immunoreceptor with Ig and ITIM domains (TIGIT), PD-1 programmed cell death 1 (PD-1), programmed cell death 1 ligand 1 (PD-L1), natural killer group 2 member A (NKG2A), V-domain immunoglobulin suppressor of T cell activation (VISTA), and immunosuppressive cytokines were quantified. The *t*-test was applied to compare the differences in the expression levels of immunomodulators and immunosuppressive cytokines between the low- and high-risk groups. A two-sided *p* < 0.05 was considered to indicate statistical significance.

## Results

### Characterization of Hypoxia-Related Genes

**Figure 2:**
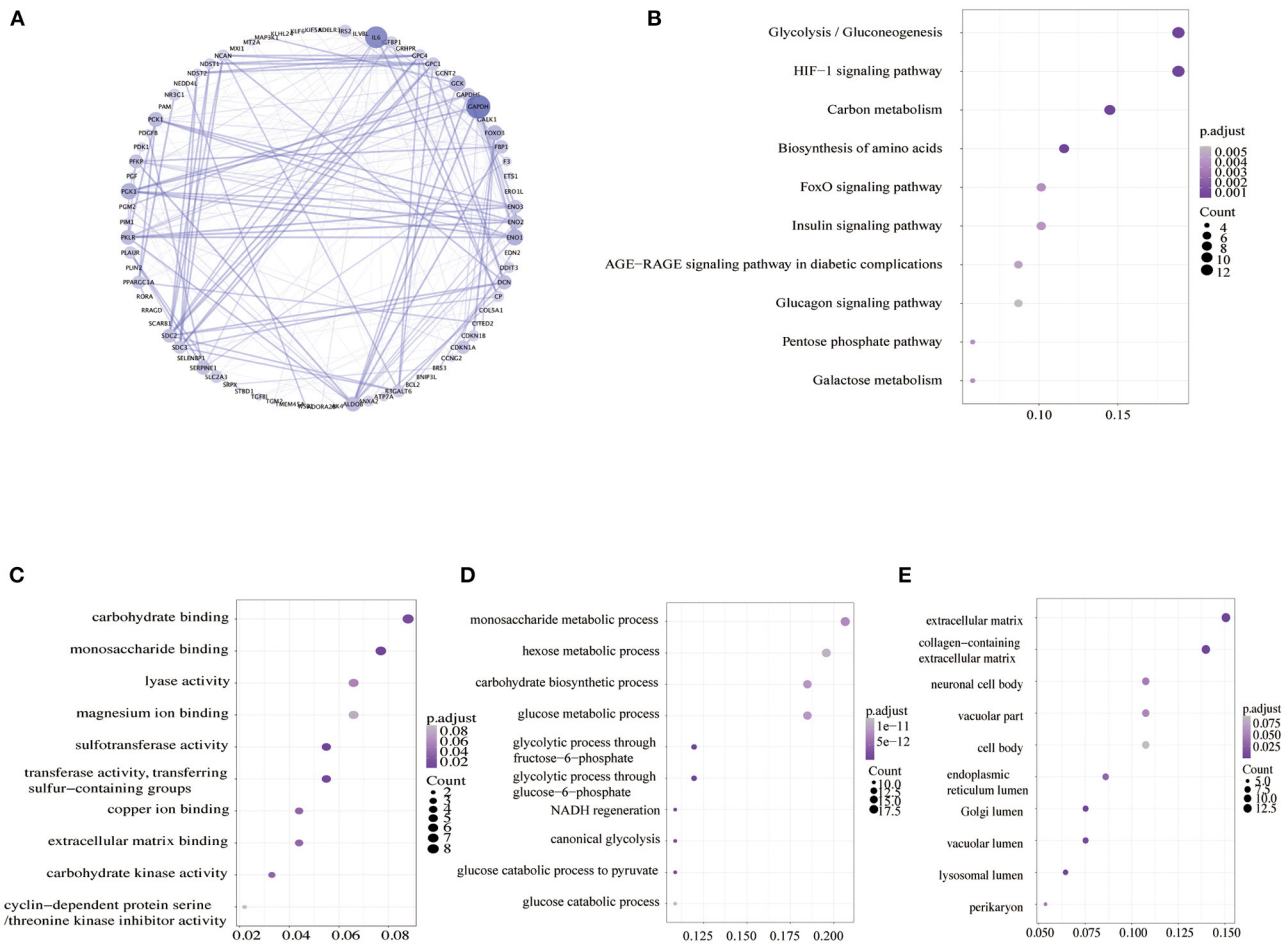
Analysis of hypoxia-related genes. **(A)** PPI networks of hypoxia-related genes. A large node means a higher degree. **(B)** KEGG pathway enrichment analysis of hypoxia-related genes. **(C–E)** GO enrichment analysis of molecular function (MF), biological process (BP), and cellular component (CC). PPI, the protein–protein interaction; GO, Gene Ontology; KEGG, Kyoto Encyclopedia of Genes and Genomes.

The hypoxia-related gene (hallmark- hypoxia genes) dataset downloaded from GSEA contained 200 genes. These genes were upregulated following treatment with low oxygen levels. In conjunction with the gene expression profiles of 526 patients with RCC downloaded from TCGA database, the prognostic predictive value of hypoxia-related genes was explored using Kaplan–Meier survival curves. Ninety-three genes were found to be significantly associated with poor overall survival outcomes according to log-rank test (*p* < 0.05; Supplemental Table 1). The STRING database and Cytoscape software were used to build the PPI network of these genes (Figure 2A). To evaluate the 93 genes, we performed KEGG and GO analyses. KEGG analysis illustrated that the genes primarily participated in 10 pathways (Figure 2B), including glycolysis/gluconeogenesis, HIF-1 signaling pathway, carbon metabolism, biosynthesis of amino acids, etc. The 285 GO terms, including 269 biological process terms, eight cellular component terms, and eight molecular function terms, were enriched (*p* < 0.05; Supplemental Table 2). The top GO terms, including carbohydrate binding, monosaccharide metabolic process, and extracellular matrix, are shown in Figures 2C–E.

### Evaluation Prognosis Prediction Power of the Hypoxia-Related Risk Model

**Figure 3:**
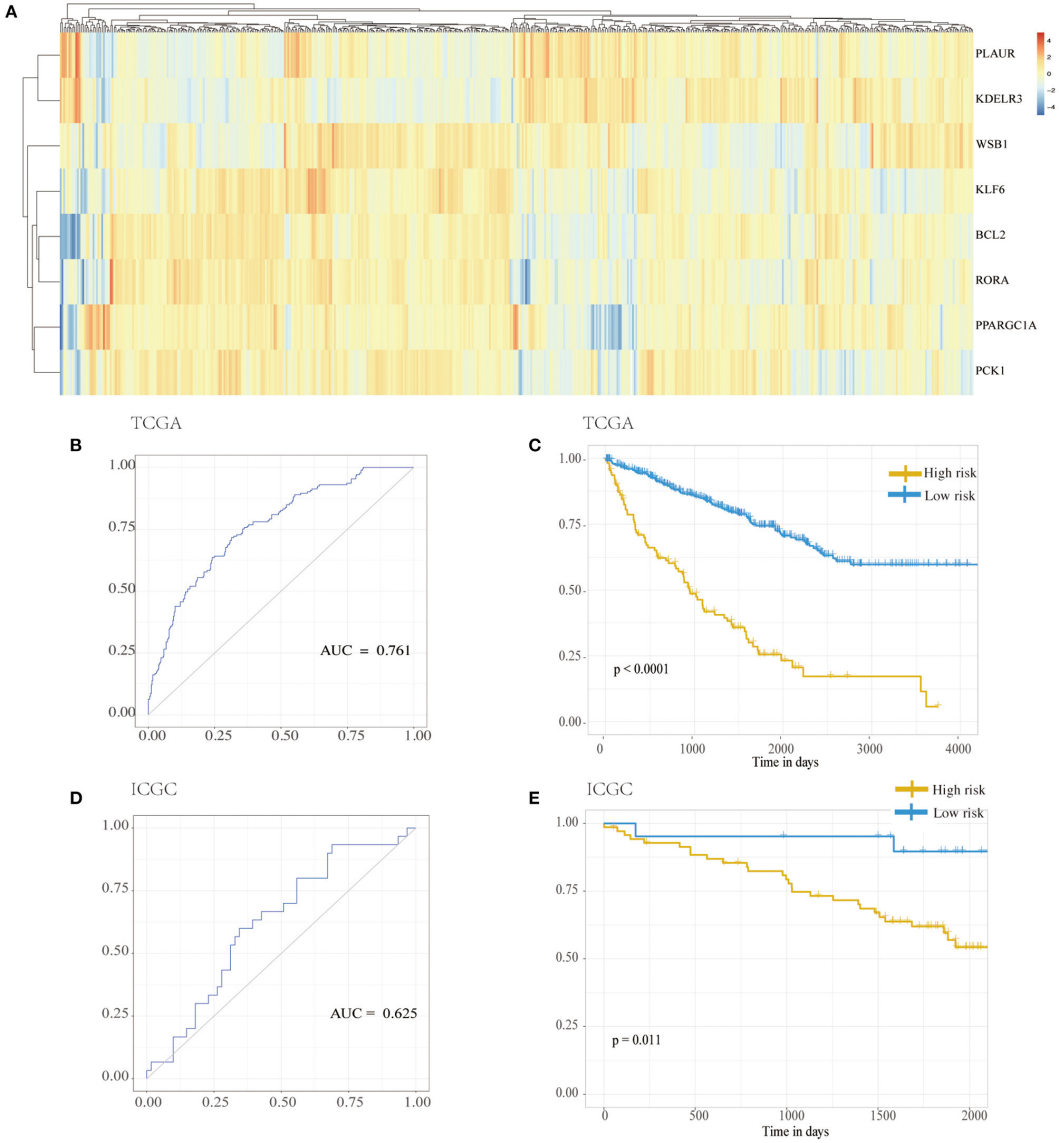
Hypoxia risk model. **(A)** Distribution of genes in the hypoxia risk model. **(B)** ROC analysis for the hypoxia risk model. **(C)** Kaplan–Meier curves for overall survival of risk score in TCGA cohort. **(D)** ROC analysis for the hypoxia risk model in the ICGC cohort. **(E)** Kaplan–Meier curves for overall survival of risk score in ICGC cohort. ICGC, International Cancer Genome Consortium; ROC, receiver operating characteristic; TCGA, The Cancer Genome Atlas.

LASSO was used to explore the hypoxia-related risk model predicting the prognosis of patients with RCC. The optimal LASSO model was selected that included eight identified genes, *PLAUR, BCL2, KLF6, KDELR3, WSB1, PPARGC1A, PCK1*, and *RORA*. The risk score was calculated using the following formula: risk score = 0.34577 × expression (*PLAUR*) + 0.18588 × expression (*BCL2*) + (−0.45209) × expression (*KLF6*) + 0.22279 × expression (*KDELR3*) + 0.53993 × expression (*WSB1*) + (−0.13366) × expression (*PPARGC1A*) + 0.01587 × expression (*PCK1*) + (−0.55309) × expression (*RORA*). Figure 3A shows the heatmap exhibiting the distinct gene expression patterns of the selected genes. Receiver operating characteristic (ROC) curves were used to evaluate the prognosis prediction power of the hypoxia-related risk model shown in Figure 3B. The model had an accuracy of 76.1% (95% CI: 0.719–0.804), and its predictive ability was higher than those of any other clinical characteristics (Table 1).

**Table 1:** The predictive accuracy of the hypoxia-related risk model and other clinical characteristics.

	**AUC**	**95% CI**
Age	62.9%	0.579–0.680
Gender	49.0%	0.447–0.534
Metastasis	62.9%	0.589–0.669
TNM staging	74.6%	0.701–0.791
Radiation therapy	48.5%	0.439–0.530
Hypoxia risk score	76.1%	0.719–0.804

Based on the chosen cutoff value of 0.5, the cases were divided into high and low hypoxia risk score group. According to the Kaplan–Meier curve, Figure 3C illustrates that the low-risk score group had a better overall survival outcome than the high-risk score group (*p* < 0.001). Adjusting for confounding variables, including age, gender, metastasis, TNM staging, and radiation therapy, Cox proportional hazards regression analysis revealed that the hypoxia risk score was an independent predictor of RCC patient prognosis, as shown in Table 2 [hazard ratio (HR) = 2.884; 95% CI: 2.090–3.979] (*p* < 0.001).

**Table 2:** The univariate analysis and multivariate analysis of the hypoxia risk score.

**Parameter**	**Univariate analysis**			**Multivariate analysis**		
	**HR**	**95% CI**	***p***	**HR**	**95% CI**	***p***
Age	1.033	(1.019–1.046)	<0.001	1.036	(1.022–1.051)	<0.001
Gender	0.941	(0.6902–1.283)	0.7	1.053	(0.767–1.446)	0.749
Metastasis	3.651	(2.68–4.974)	<0.001	1.503	(0.987–2.289)	0.058
TNM staging	1.856	(1.629–2.115)	<0.001	1.482	(1.241–1.770)	<0.001
Radiation therapy	0.775	(0.574–1.046)	0.095	0.862	(0.636–1.167)	0.336
Hypoxia risk score	4.315	(3.185–5.845)	<0.001	2.884	(2.090–3.979)	<0.001

From the ICGC database, data from a cohort of 91 patients with RCC was obtained to verify the results. As shown in Figure 3D, the accuracy of the model was 62.5% (95% CI: 0.505–0.745) in the validation samples. Additionally, Figure 3E also shows that the low-risk score group had a better overall survival outcome compared to the high-risk score group (*p* = 0.011).

### Immune Landscape of High/Low Hypoxia Risk Score Groups

**Figure 4:**
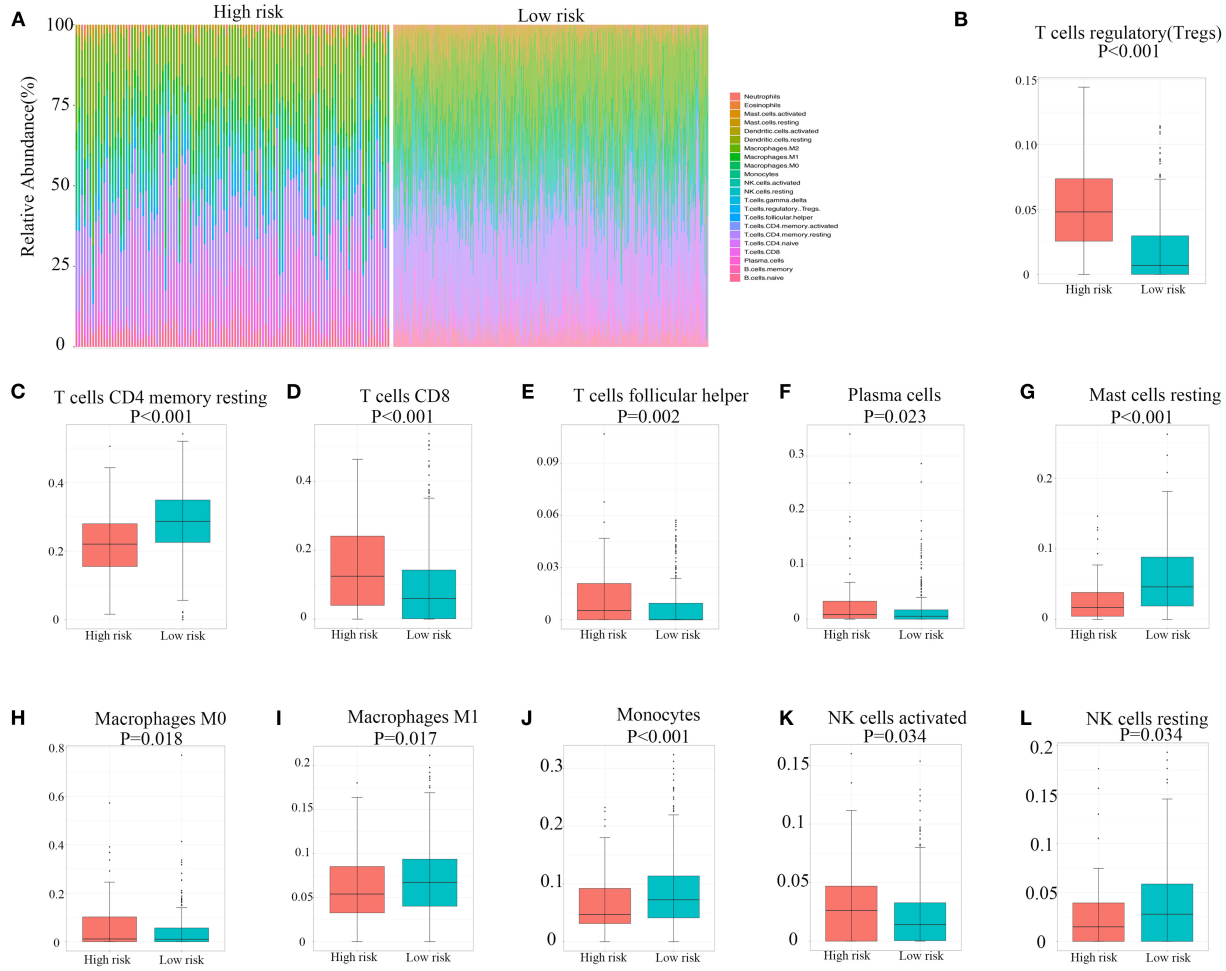
Immune landscape between high and low hypoxia risk score groups of renal cell carcinoma patients. **(A)** The abundance of immune infiltration in high-risk and low-risk groups from TCGA cohort. **(B–L)** The proportions of different immune cells between high-risk and low-risk groups in TCGA cohort. TCGA, The Cancer Genome Atlas.

The capability of the hypoxia-related risk model to assess the immune microenvironment of RCC was evaluated. We utilized the CIBERSORT method with the LM22 signature gene file to assess the immune cell fraction between the low- and high-risk groups. A summary of the results based on 526 patients with RCC downloaded from TCGA database is illustrated in Figure 4A. The proportions of immunosuppressive cells, such as Tregs, were significantly higher in the high hypoxia risk score groups, as shown in Figure 4B. This indicates that patients with high hypoxia risk scores possess an immunosuppressive microenvironment. Figures 4C–L show other types of immune cells, which exhibited significantly different proportions between the low- and high-risk groups.

### Expression Profile of Immunomodulators and Immunosuppressive Cytokines

**Figure 5:**
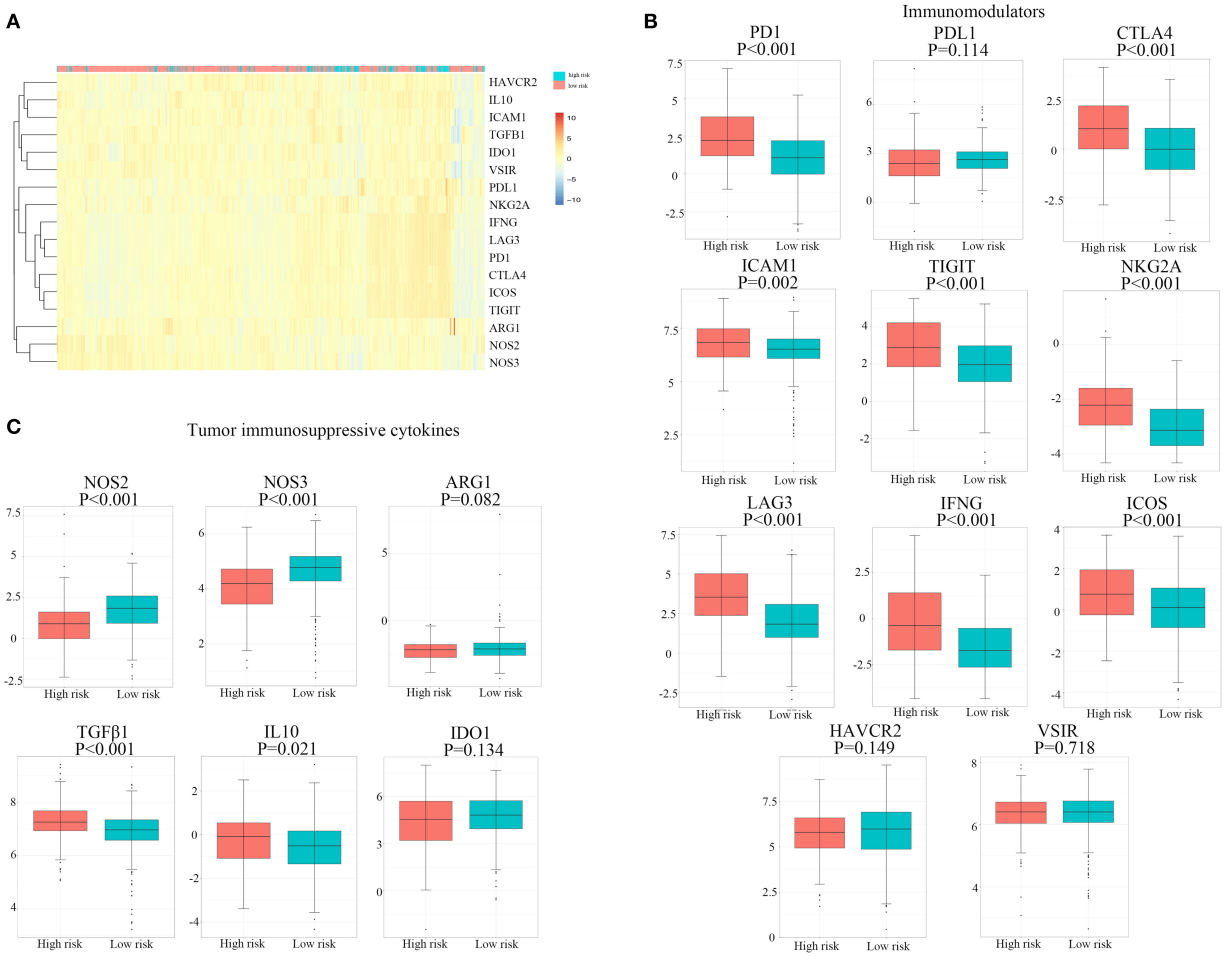
Expression profile of immunomodulators and immunosuppressive cytokines. **(A)** Distribution of genes of immunomodulators and immunosuppressive cytokines. **(B,C)** The proportions of immunomodulators and immunosuppressive cytokines between high and low hypoxia risk groups in TCGA cohort. TCGA, The Cancer Genome Atlas.

The expression of 11 immunomodulators and six immunosuppressive cytokines in 526 patients with RCC downloaded from TCGA database is illustrated in Figure 5A. We found that the expression of PD-1, CTLA-4, ICAM-1, TIGIT, NKG2A, LAG-3, IFNG, and ICOS was significantly upregulated in the high hypoxia risk score group, as shown in Figure 5B. Immunosuppressive cytokines, such as transforming growth factor (TGF)-β1 and IL-10, were also significantly upregulated in the high hypoxia risk score group, as shown in Figure 5C. However, NOS2 and NOS3 were significantly reduced in the high-risk group. As a result, patients with RCC in the high hypoxia risk score group may have an immunosuppressive tumor microenvironment with upregulated immunomodulators and immunosuppressive cytokines. Therefore, targeting hypoxia may benefit immunotherapy in clinical practice.

## Discussion

Previous studies demonstrate that hypoxia and hypoxia-related signaling pathways play important roles in the development and progression of RCC (Schödel et al., 2016). Von Hippel–Lindau tumor suppressor (pVHL) and HIFs are critical factors in these pathways. With tumor cell proliferation and growth, RCC results in hypoxia with the activated HIF-α signaling in response to oxygen deprivation (Millet-Boureima et al., 2021). On the other hand, the VHL gene is lost in ~90% of ccRCC tumors (Linehan and Ricketts, 2019). In normal renal tissue, oxygen-dependent posttranslational modifications on HIF-2α allow pVHL to normally recognize and mediate the proteasomal degradation (Choueiri and Kaelin, 2020). Loss of VHL gene in ccRCC, tumor is under pseudohypoxia state with accumulated HIF-2α and activated HIF-1 to upregulate the expression of hypoxia-inducible genes and increase tumor oxygenation (Haase, 2013). Hypoxia is a phenomenon in other cancers. HIF-2α has been known to regulate tumor proliferation, metabolism, metastasis, and resistance to chemotherapy in digestive system cancers (Zhao et al., 2015). In melanoma, a hypoxia-related signature has been developed to predict prognosis (Shou et al., 2021).

In the present study, we identified 93 hypoxia-related genes significantly associated with the outcomes of patients with RCC. The PPI network of these selected genes significantly included glyceraldehyde-3-phosphate dehydrogenase (GAPDH), IL-6, phosphoglycerate kinase 1 (PGK1), enolase 1 (ENO1), and glucokinase (GCK). GAPDH is a key enzyme involved in glycolysis and is related to cell proliferation in RCC (Vilà et al., 2000). IL-6 has been shown to induce drug resistance in RCC and is associated with poor prognosis (Ishibashi et al., 2018). PGK1 is also a glycolytic enzyme that can be secreted by tumor cells to participate in angiogenesis. ENO1, GCK, PGK1, and GAPDH are involved in tumor energy metabolism. Functional enrichment analysis revealed that these genes were specifically related to the glucometabolic process, hypoxia-related pathway, carbon metabolism, and extracellular matrix. These results suggest that hypoxia-related energy metabolism is associated with tumor prognosis and the TME condition.

Multiple approaches have been developed to predict the prognosis of RCC, including prognostic models and nomograms. Tumor node metastasis classification remains the most important identified prognostic factor (Klatte et al., 2018). Immunohistochemical staining of Ki-67, p53, and VEGFR-1 was shown to be significantly related to RCC outcomes. Molecular markers have also been applied as prognostic models. The ClearCode34-based model was developed including 34 genes to classify the subtypes of localized ccRCC to predict patient survival outcomes (Brooks et al., 2014). The continuous CLEAR score (continuous linear enhanced assessment of ccRCC) was developed based on an 18-transcript signature to predict patients' disease-specific survival and the response to tyrosine kinase inhibitor (Wei et al., 2017).

We developed a hypoxia-related risk model based on hypoxia-related genes to predict the prognosis of patients with RCC. The model had an accuracy of 76.1% (95% CI: 0.719–0.804) and was found to be an independent predictor in Cox proportional hazards regression analysis. *PLAUR* encodes the receptor for urokinase plasminogen activator. *BCL2* encodes a membrane protein that regulates lymphocyte apoptosis. *KLF6* encodes the zinc finger protein that acts as a tumor suppressor. *KDELR3* encodes a member of the KDEL endoplasmic reticulum protein retention receptor family. *WSB1* encodes a member of the WD-protein subfamily. *PPARGC1A* encodes proteins that regulate energy metabolism. *PCK1* is a critical regulator of gluconeogenesis. *RORA* participates in tumor metastasis regulation. *PLAUR, BCL2, KLF6, WSB1, PPARGC1A*, and *PCK1* were identified to be related to the prognosis of ccRCC (Hirata et al., 2009; Syafruddin et al., 2019; Xu et al., 2019; Liu et al., 2020; Shen et al., 2020; Shi et al., 2020). The functions of KDELR3 and RORA have not been reported in RCC.

Further analysis demonstrated that the hypoxia-related risk model was also related to the immune microenvironment of RCC. Tregs are key players in tumor immune escape and angiogenesis (Facciabene et al., 2012). Monocytes congregate in the TME and differentiate into tumor-associated macrophages (TAMs). Hypoxia has a profound effect on these cells (Lewis and Murdoch, 2005). Tregs were discovered to be significantly higher in the high hypoxia risk score groups, indicating an immunosuppressive microenvironment in these patients. Monocytes and M1 macrophages, which can function as efficient immune effector cells and promote antitumor immune responses, were suppressed in patients with high hypoxia risk scores. Additionally, our results showed that PD-1 and CTLA-4 were significantly upregulated in the high hypoxia risk score groups. However, CD8^+^ T cells and activated natural killer cells were higher in the high hypoxia risk score groups. These results indicate that hypoxia condition has multiple effects on the immune microenvironment. The hypoxia-related risk model may be useful for predicting the immunotherapy response. Improving oxygen deficiency may decrease immunosuppression in the TME of RCC, which may benefit immunotherapy.

It remains difficult to predict or explain the clinical response rate of RCC immunotherapy in practice. However, hypoxia has been reported to lead to immunosuppression and tumor progression (Li et al., 2018). Hypoxia-induced changes in the TME have also been reported as a barrier to immunotherapy in pancreatic adenocarcinoma (Daniel et al., 2019). Furthermore, inhibition of hypoxic stress-relevant pathways can enhance antitumor immunity and improve the response rate of immunotherapy. mTOR inhibitors are applied in current therapies for RCC to target HIF translation. VEGFA inhibitors target the function of HIF-target genes. This may help prevent drug resistance and enhance immunotherapy.

There were some limitations to this study. First, the results are based on data collected from TCGA database. Although the results were verified in the ICGC database with 91 cases, the potential for selection bias cannot be avoided, and it is impossible to collect all clinical information from the patients. Second, the results are descriptive, and *in vitro* or *in vivo* experiments were not performed to clarify the exact immune microenvironment of RCC. Third, further clinical trials are needed to validate the prognostic prediction power of the hypoxia-related risk model. Last but not least, the comparison between different tools for predicting the prognosis of RCC, which may lead to a more objective evaluation of the novel hypoxia-related risk model, is not included in our study. Despite these limitations and lack of further validation in more studies, the presented findings applied a hypoxia-related risk model in RCC prognosis predicting and statistically proved its performance.

We developed a hypoxia-related risk model based on eight identified hypoxia-related genes. The model was validated as an independent predictor of the prognosis of patients with RCC. We hope that the hypoxia-related risk model can be used as a prognostic biomarker in patients with RCC, which may be helpful for underpinning clinical decision-making in the future. Moreover, the hypoxia-related risk model may reflect the immune microenvironment of RCC and help improve the overall clinical response to immunotherapy.

## Data Availability Statement

The original contributions generated for the study are included in the article/Supplementary Material, further inquiries can be directed to the corresponding author/s.

## Author Contributions

YM and QZ contributed to the design and supervision of the study and contributed to manuscript revision. ZZ and QL contributed to the online data search, acquisition, interpretation, and contributed to manuscript writing. FW and BM contributed to the data extraction. All authors made substantial contributions to the study and have approved the study and are responsible for their own contributions to the study personally.

## Conflict of Interest

The authors declare that the research was conducted in the absence of any commercial or financial relationships that could be construed as a potential conflict of interest.
